# Topical latanoprost acid for female androgenetic alopecia: a pilot proof-of-concept trial with mechanistic evidence of prostaglandin F2α receptor activation

**DOI:** 10.1007/s43440-026-00876-0

**Published:** 2026-07-10

**Authors:** Adriana Rakowska, Małgorzata Dutkiewicz, Oliwia Zegrocka-Stendel, Dorota Dymkowska, Grzegorz Huszcza, Maciej Wierzbicki, Jarosław Walczak, Lidia Rudnicka, Katarzyna Koziak

**Affiliations:** 1 Department of Dermatology, Medical University of Warsaw, Warszawa, Poland; 2 Department of Biochemistry and Nutrition, Centre for Preclinical Research and Technologies, Medical University of Warsaw, ul. Banacha 1b, Warszawa, 02-097 Poland; 3 Laboratory of Cellular Metabolism, Nencki Institute of Experimental Biology, Polish Academy of Sciences, Warszawa, Poland; 4 Department of Pharmacy, Pharmaceutical Research Institute, Warszawa, Poland; 5 BioResearch Pharma S.A., Warszawa, Poland; 6 Department of Biosystems and Soft Matter, Institute of Fundamental Technological Research, Polish Academy of Sciences, Warszawa, 02-106 Poland

**Keywords:** Androgenetic alopecia, Latanoprost acid, Prostaglandin F2α receptor, Dermal papilla, Calcium signaling, Hair growth

## Abstract

**Background:**

Prostaglandin F2α receptor (FP receptor) signaling is a plausible target for promoting hair growth, but clinical data on topical latanoprost acid (the active free-acid FP agonist) in hair loss are lacking. This study aimed to evaluate the clinical efficacy, safety, and mechanistic basis of topical latanoprost acid in women with female androgenetic alopecia.

**Methods:**

In this investigator-initiated, randomized, double-blind, single-center, dose-ranging pilot trial, 29 adult women with hair loss predominantly consistent with female androgenetic alopecia were randomized to vehicle (*n* = 2) or topical latanoprost acid 0.01% (*n* = 8), 0.05% (*n* = 13), or 0.1% (*n* = 6), applied once daily for 6 months. The primary endpoint was within-participant change in target-area hair count (TAHC, hairs/cm²) from baseline to month 6; trichoscopic activity markers (yellow dots) and follicular-unit (FU) remodeling were secondary and exploratory outcomes. Human hair dermal papilla cells (HHDPCs) were assessed for FP receptor-linked signaling (intracellular Ca²⁺ flux) and DNA synthesis by 5-ethynyl-2′-deoxyuridine (EdU) incorporation after exposure to latanoprost acid versus equimolar latanoprost.

**Results:**

An increase in TAHC was observed across all active treatment arms (mean ± SEM ΔTAHC: 17.8 ± 4.3, 23.5 ± 6.1, and 16.5 ± 6.5 hairs/cm² in the latanoprost acid 0.01%, 0.05%, and 0.1% arms, respectively). No significant between-arm differences were detected. Secondary and exploratory trichoscopic analyses showed reductions in yellow-dot counts, a decrease in single-hair FUs, and an increase in triple-hair FUs. Safety was favorable, with no serious adverse events. In mechanistic assays, latanoprost acid triggered rapid, concentration-dependent Ca²⁺ flux, whereas equimolar latanoprost produced delayed signals; neither compound altered EdU incorporation.

**Conclusions:**

In this pilot proof-of-concept trial, topical latanoprost acid showed a coherent clinical-trichoscopic bioactivity signal, supported by FP receptor-linked signaling in HHDPCs. These findings require confirmation in larger randomized pharmacokinetic/pharmacodynamic-integrated trials designed to optimize dose, confirm efficacy, and further characterize long-term safety.

**Trial registration:**

ClinicalTrials.gov, NCT07412587; registered on February 2, 2026.

**Supplementary Information:**

The online version contains supplementary material available at 10.1007/s43440-026-00876-0.

## Introduction

Hair loss is a common condition affecting both men and women and is frequently associated with distress, reduced self-esteem, and diminished quality of life [1, 2]. In women, female androgenetic alopecia is the most common cause of the female pattern hair loss phenotype. It manifests as diffuse thinning over the vertex and mid-scalp, whereas chronic telogen effluvium presents with persistent diffuse shedding [1, 3]. Existing therapies, including topical or oral minoxidil and oral anti-androgens (e.g., finasteride, spironolactone, or dutasteride), can be beneficial for many patients. However, treatment responses vary, and long-term use may be limited by tolerability, contraindications, or patient preference, underscoring the need for additional safe and effective options [4–6].

Since the initial observation of eyelash hypertrichosis in glaucoma patients treated with latanoprost, prostaglandin analogues have been recognized as promising hair-growth modulators [7]. Prostaglandin signaling provides a biologically plausible mechanism for promoting hair growth [8]. In the follicle, prostaglandin F2α receptor (FP receptor) is expressed in anagen human scalp hair follicles, including dermal papilla and perifollicular compartments [8]; expression in non-scalp body hair follicles has not been systematically characterized. The receptor signals via Gq-coupled phosphoinositide/phospholipase C pathways, leading to intracellular Ca²⁺ mobilization [9]. Clinically, FP-pathway agonism has been associated with increased hair growth and pigmentation in clinical dermatologic contexts [7, 10]. Despite this receptor-level rationale, clinical work to date has centered on marketed prostaglandin analogues used in glaucoma, particularly topical latanoprost and, to a lesser extent, bimatoprost/prostamide analogues. Latanoprost is administered as an isopropyl ester prodrug that undergoes hydrolysis to latanoprost acid, the active FP receptor agonist. Consequently, direct evaluation of latanoprost acid itself as the active free-acid form in scalp hair loss remains largely unexplored [11].

Given that prostaglandin analogue activation depends on tissue hydrolases (e.g., esterases for latanoprost; amidases generating the carboxylic acid from bimatoprost) [12, 13] and that cutaneous drug-metabolizing enzyme activity can vary by anatomical site and between individuals [14, 15], direct testing of active moieties could yield more predictable target engagement. Moreover, as a charged free acid, latanoprost acid is expected to have lower trans-barrier flux, which may limit systemic exposure with topical scalp application [16, 17]. Latanoprost was explicitly designed as an isopropyl ester prodrug to enhance tissue penetration, followed by hydrolysis to latanoprost acid after barrier crossing [12, 16]. Consistent with this principle, ocular dosing with latanoprost has been associated with detectable systemic exposure to latanoprost acid [16]. These considerations support evaluating latanoprost acid itself as a topical scalp treatment.

Here, we report the first, to our knowledge, investigator-initiated clinical evaluation of topical latanoprost acid in women with hair loss. We assessed changes in target-area hair count (TAHC) and trichoscopic outcomes, and we complemented clinical findings with mechanistic experiments in human hair dermal papilla cells (HHDPCs), including FP receptor engagement (Ca²⁺ flux), DNA synthesis, and metabolic activity assays following exposure to latanoprost acid versus latanoprost.

## Materials and methods

### Clinical study

#### Study design, participants, and ethics

This investigator-initiated, randomized, double-blind, single-center, dose-ranging pilot trial was conducted at the Department of Dermatology, Medical University of Warsaw. Participants received once-daily topical treatment for 6 months, with evaluations at baseline, month 3, and month 6. The study protocol was approved by the Ethics Committee of the Medical University of Warsaw (KB/148/2015; approved on July 7, 2015). The privacy rights of participants were observed. All participants provided written informed consent before enrollment. The study was conducted in accordance with the Declaration of Helsinki and the principles of Good Clinical Practice. The study was registered at ClinicalTrials.gov (Identifier: NCT07412587; first posted on February 2, 2026).

Women aged 18–60 years with patterned scalp hair loss consistent with female androgenetic alopecia (Ludwig I–III) or chronic telogen effluvium were eligible. For participants with female androgenetic alopecia, the diagnosis was confirmed by trichoscopy [18]. Exclusion criteria included recent use of hair-growth treatments, active scalp dermatoses, or significant uncontrolled medical conditions. Forty patients were screened; 29 were enrolled.

#### Randomization, masking, and interventions

Participants were randomized to vehicle or latanoprost acid at one of three concentrations (0.01%, 0.05%, or 0.1%) in identical, coded dropper bottles. Allocation was computer-generated, and masking was maintained for participants and investigators through month 6. The assigned study product was applied once daily to the androgen-dependent scalp region (i.e., the clinically affected area). Participants were instructed to apply 2–3 mL as needed to cover the area, distribute it evenly, avoid runoff, and refrain from washing the area for ≥ 8 h. The tested product was formulated as an oil-in-water (o/w) emulsion. Major excipients included water, phosphate buffer, EDTA, xanthan gum, paraffin, an emulsifying system based on a mixture of fatty alcohols and their ethers, propylene glycol, and phenoxyethanol. The placebo vehicle was identical, without latanoprost acid. Hair-care routines were kept constant; concomitant hair-loss therapies were prohibited. Clinical and trichoscopic outcome assessments were performed at standardized assessment sites/fields at fixed scalp locations within the treated region, using the same locations consistently across visits.

#### Outcomes

The primary endpoint was the within-subject absolute change in TAHC (ΔTAHC, hairs/cm²) from baseline to month 6 (month 3 change supportive). Percent change in TAHC (%TAHC) from baseline was summarized as a supportive analysis. Secondary endpoints included trichoscopic features (hair-shaft thickness grade 0–3, follicular-unit (FU) arrangement, number of yellow dots) and patient-reported outcomes. Safety/tolerability endpoints comprised adverse events, local scalp findings (erythema/irritation), and vital signs.

#### Assessments

Standardized clinical macrophotographs of the androgen-dependent scalp region and trichoscopy were obtained at each visit using a consistent acquisition protocol. Trichoscopy images were acquired at fixed scalp locations within the treated region (20× and 70× magnification). For TAHC, assessments were performed at a fixed site within the androgen-dependent scalp region along the midline, 1 cm from the frontal hairline, according to a standardized trichoscopic procedure. The same predefined area was evaluated at each visit. TAHC was determined by applying a 1 cm² counting frame to the 20× trichoscopy image and manually counting all visible hairs within the frame. No tattoo or micro-dot marking was used. Trichoscopic images used for TAHC and other trichoscopic analyses were evaluated without reference to treatment allocation labels; image coding and manual counting were performed by the same assessor. Trichoscopy also captured FU arrangement (percentage of FUs with single hair [%FU1] and with three hairs [%FU3]) and the number of yellow dots, assessed from images obtained at the same standardized locations. Patient self-assessments (shedding, thickness, overall growth) were collected at months 3 and 6 using a structured questionnaire. Safety was assessed at each visit via systematic adverse-event queries, targeted scalp examination, and vital signs.

### In vitro experiments

Functional effects of latanoprost acid and latanoprost on HHDPCs were evaluated using complementary assays addressing receptor signaling and proliferation-related cellular responses. Ca²⁺-flux assays measured intracellular calcium dynamics as a direct marker of FP receptor activation, while EdU incorporation and PrestoBlue™ assays assessed DNA synthesis and metabolic activity, respectively.

#### Intracellular Ca²⁺ flux

Primary HHDPCs (Innoprot, Spain) were cultured in Mesenchymal Stem Cell Medium with growth supplement and penicillin/streptomycin on poly-L-lysine-coated plastic (0.01% for 30 min). Cells were seeded on glass coverslips, grown for 48 h to confluence, and loaded with Fura-2 AM (4 µM, 30 min, 37 °C). Ratiometric cytosolic [Ca²⁺] signals (F340/F380) were recorded at RT in physiological buffer (with 2 mM CaCl₂) using a spectrofluorimeter (Hitachi F-7000 or equivalent). Latanoprost acid and latanoprost were applied at concentrations ranging from 31.25 to 500 nM; vehicle controls were included (0 nM).

#### DNA synthesis assay

DNA synthesis was quantified using the 5-ethynyl-2’-deoxyuridine (EdU) Proliferation Kit (Lumiprobe) according to the manufacturer’s instructions. Cells were seeded in poly-L-lysine-coated 96-well optical-bottom plates (Greiner) at a density of 1,600 cells per well. HHDPCs were exposed to latanoprost acid or latanoprost in complete medium and assessed after 24, 48, or 72 h. Concentrations ranged from 62.5 to 1000 nM at 24 h and from 31.25 to 1000 nM at 48 h and 72 h. Cells were pulsed with EdU (10 µM, 1 h), fixed with 4% paraformaldehyde, permeabilized (0.5% Triton X-100), and stained via copper(I)-catalyzed azide–alkyne click chemistry with sulfo-Cy3–conjugated azide. Nuclei were counterstained with Hoechst 33,342. Images were acquired with a 20× objective on the Operetta CLS imaging system under identical settings. At least 13 random fields per condition per experiment were analyzed with Harmony 4.9 software. The percentage of EdU-positive nuclei was calculated as the ratio of EdU-positive (Cy3-positive) nuclei to total Hoechst-positive nuclei.

#### PrestoBlue™ metabolic activity assay

Metabolic activity of HHDPCs was assessed using the PrestoBlue™ (Thermo Fisher Scientific/Invitrogen) resazurin-based assay according to the manufacturer’s instructions. Cells were exposed to vehicle or the indicated concentrations of latanoprost acid or latanoprost, followed by incubation with PrestoBlue™ reagent and measurement of fluorescence. Signals were background-subtracted and normalized to vehicle controls (0 nM).

### Statistical analysis

All analyses were exploratory. Continuous outcomes are presented as mean ± SEM unless stated otherwise. Repeated-measures trichoscopic efficacy endpoints, including TAHC, ΔTAHC, %TAHC, yellow-dot counts, and FU arrangement measures, were analyzed using a mixed-effects model for repeated measures fitted by restricted maximum-likelihood (REML), with time, treatment, and their interaction as fixed effects, and participant as a random effect. For outcomes expressed as change from baseline (e.g., ΔTAHC and %TAHC), the dependent variables were the change scores at month 3 and month 6. Use of change-from-baseline scores as the outcome inherently adjusts for individual baseline values within each participant and is a standard approach to handling baseline imbalance in repeated-measures designs. This approach uses all available observations without imputing missing values. Treatment-specific post hoc comparisons were interpreted only when the time × treatment interaction was significant and were performed using Tukey’s multiple comparisons test. Exploratory baseline-adjusted sensitivity analyses were performed for month 6 trichoscopic efficacy outcomes using multiple linear regression, with the month 6 value as the dependent variable, the corresponding baseline value as a covariate, and treatment arm as a categorical predictor. Analyses were performed in participants with available baseline and month 6 data for the corresponding endpoint. Results are reported as overall model effects, baseline covariate slopes with 95% confidence intervals, baseline-adjusted treatment-arm effects, and model R². Exploratory responder analyses based on month 6 ΔTAHC were performed using Fisher’s exact test for contingency tables. For mixed-effects models, post hoc results, when reported, are presented as model-estimated least-squares mean differences with 95% confidence intervals and Tukey-adjusted p-values. For trichoscopic outcomes analyzed on the raw scale (yellow dots, %FU1, %FU3), baseline was included as a time point within the repeated-measures model, allowing the model to account for baseline variation between treatment arms. The investigator-rated hair-shaft thickness improvement grade (ordinal scale 0–3) assessed at month 6 was compared across treatment arms using the Kruskal–Wallis test. Patient-reported overall improvement was summarized as n/N (%) at month 3 and month 6 and compared across treatment arms using Fisher’s exact test for contingency tables; these analyses were exploratory and not adjusted for multiplicity. For in vitro EdU incorporation experiments, the percentage of EdU-positive nuclei was averaged across technical replicates within each independent experiment before statistical analysis. Each exposure time point was analyzed separately. In each independent experiment, latanoprost acid and latanoprost were considered as separate treatments, with concentration representing dose levels within each treatment. Accordingly, concentration series were analyzed separately for each compound using one-way ANOVA. Dunnett’s multiple comparisons versus the corresponding vehicle control (0 nM) was used for post hoc comparisons when applicable. All tests were two-sided; *p* < 0.05 was considered statistically significant. Analyses were performed in GraphPad Prism (version 10.2; GraphPad Software, San Diego, CA, USA).

## Results

### Clinical findings

#### Participant disposition and baseline characteristics

Twenty-nine women were randomized to receive vehicle or one of three latanoprost acid concentrations: 0.01%, 0.05%, or 0.1%. Recruitment was discontinued before the planned sample size of 40 participants was reached. As a result, the treatment arms had unequal sample sizes. Of the 29 randomized women, 25 participants (86%) completed the month 3 visit, and 24 participants (83%) completed the month 6 visit. Five participants did not complete the study. In the 0.01% arm, two participants were lost to follow-up after the baseline visit, and one participant discontinued the study at month 3 because of headache. In the 0.05% arm, one participant withdrew approximately 4 weeks after randomization because of increased shedding, and one participant was lost to follow-up after the baseline visit. The modified intent-to-treat efficacy population comprised all randomized participants with at least one post-baseline efficacy assessment; the safety population included all treated participants.

The baseline demographic and clinical characteristics of the randomized women were generally comparable across treatment arms, although selected trichoscopic variables varied numerically (Table 1). The mean age was in the mid-30s, and all participants were female. Most participants had female androgenetic alopecia, predominantly Ludwig grade I–II, and two participants had chronic telogen effluvium. Numerically, the lowest mean TAHC, the highest mean %FU1, and the lowest mean %FU3 were observed in the 0.05% arm. All efficacy analyses were conducted as within-subject changes from baseline, which accommodates between-arm baseline differences.

**Table 1:** Baseline characteristics of the study population^1^

Characteristic	Vehicle (*n* = 2)	Latanoprost acid treatment arms			Total† (*n* = 29)
		0.01% (*n* = 8)	0.05% (*n* = 13)	0.1% (*n* = 6)	
Demographics					
Age, years, mean ± SD	33.5 ± 9.2	31 ± 5.9	36.8 ± 10.6	39.3 ± 11.2	35.5 ± 9.6
Age range, years	27–40	24–41	24–60	28–59	24–60
Disease duration, years, mean ± SD	4.5 ± 3.5	3.9 ± 2.5	8.5 ± 5.1	6.1 ± 5.3	6.5 ± 4.7
Diagnosis					
FAGA, n (%)	1 (50)	8 (100)	13 (100)	5 (83.3)	27 (93.1)
Chronic telogen effluvium, n (%)	1 (50)	0 (0)	0 (0)	1 (16.7)	2 (6.9)
Ludwig grade, n (%)*					
Grade I	2 (100)	4 (50)	1 (8.3)	4 (66.7)	11 (39.3)
Grade II	0 (0)	4 (50)	6 (50)	1 (16.7)	11 (39.3)
Grade III	0 (0)	0 (0)	5 (41.7)	1 (16.7)	6 (21.4)
Baseline trichoscopic parameters					
TAHC, hairs/cm², mean ± SD	156 ± 15.6	175.5 ± 65.4ᵃ	138 ± 34.1ᵇ	165 ± 62.3	—
%FU1, %, mean ± SD	20 ± 14.1	21.9 ± 14.6	55.9 ± 29.2ᵇ	26.7 ± 36.1	—
%FU3, %, mean ± SD	20 ± 14.1	30 ± 21.4	6.4 ± 15ᵇ	41.7 ± 30.6	—
Yellow dots, mean ± SD	6.5 ± 0.7	6.4 ± 4.6	9.6 ± 4.5ᵇ	7.5 ± 6.7	—

Participants were women with hair loss predominantly consistent with female androgenetic alopecia and were randomized to vehicle or topical latanoprost acid 0.01%, 0.05%, or 0.1%, applied once daily for 6 months. Data are shown as mean ± SD or n (%), as appropriate. No formal statistical comparisons were performed for baseline characteristics

†Baseline demographic and clinical data were available for all 29 randomized participants

ᵃBaseline TAHC was available for 6 participants in the 0.01% arm

ᵇBaseline trichoscopic variables were available for 11 participants in the 0.05% arm

*Ludwig grade was available for 12 participants in the 0.05% arm

^1^Investigator-initiated, randomized, double-blind, single-center, dose-ranging pilot trial conducted in 2015–2016 at the Department of Dermatology, Medical University of Warsaw, Warsaw, Poland

Abbreviations: FAGA, female androgenetic alopecia; n, number of participants; SD, standard deviation; TAHC, target-area hair count; %FU1, percentage of single-hair follicular units; %FU3, percentage of triple-hair follicular units

#### Primary efficacy outcome: absolute change in target-area hair count (ΔTAHC)

ΔTAHC was analyzed at month 3 and month 6 as the primary measure of within-participant hair-count change during topical latanoprost acid treatment.

**Fig. 1:**
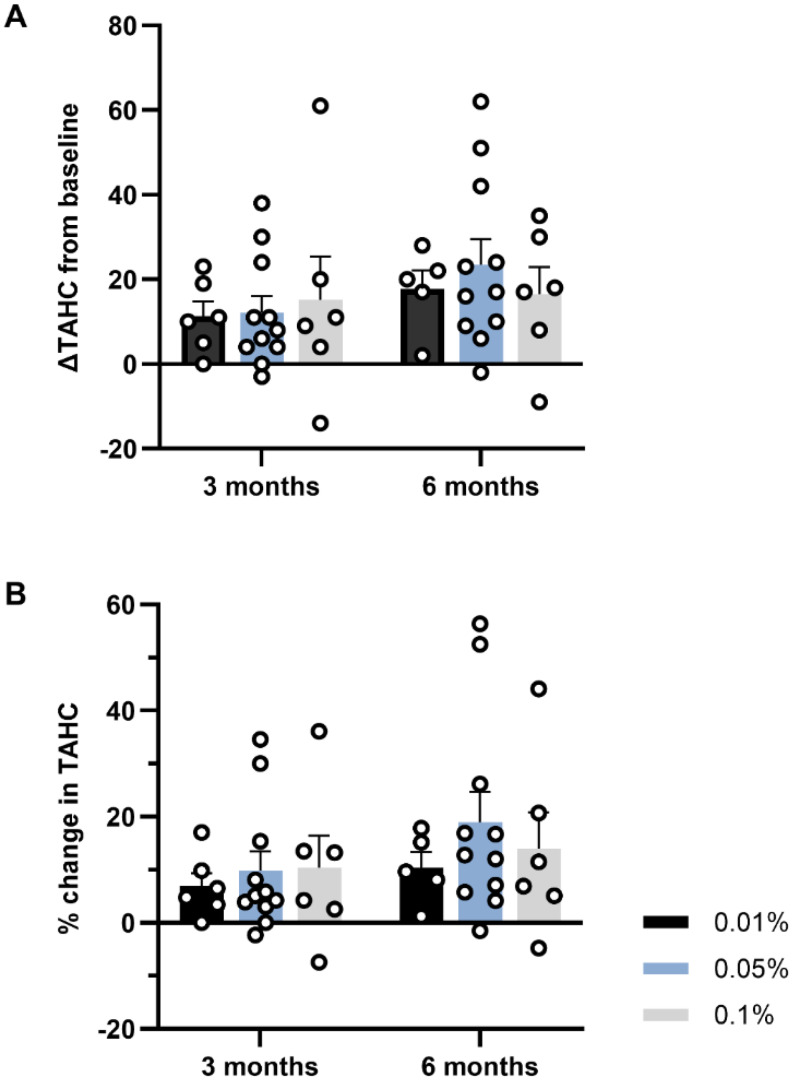
Changes in TAHC from baseline after 3 and 6 months of treatment with latanoprost acid at concentrations of 0.01%, 0.05%, or 0.1% in women with female androgenetic alopecia^1^. TAHC was assessed by manual counting of all visible hairs within a 1 cm² area delineated on standardized 20× trichoscopy images obtained at fixed scalp locations. (**A**) Absolute change from baseline (ΔTAHC, hairs/cm²). (**B**) Percent change from baseline (%TAHC). Analyses were performed in participants with available post-baseline assessments: 0.01% (*n* = 6 at month 3; *n* = 5 at month 6), 0.05% (*n* = 11 at month 3 and month 6), and 0.1% (*n* = 6 at month 3 and month 6). Results are presented as observed means ± SEM. Each point represents data from an individual patient. Statistical analysis: mixed-effects model for repeated measures (REML). Abbreviations: REML, restricted maximum-likelihood; SEM, standard error of the mean; TAHC, target-area hair count; ΔTAHC, absolute change in TAHC; %TAHC, percent change in TAHC. ^1^Investigator-initiated, randomized, double-blind, single-center, dose-ranging pilot trial conducted in 2015–2016 at the Department of Dermatology, Medical University of Warsaw, Warsaw, Poland

A mixed-effects model for repeated measures (REML) revealed a significant effect of time (F₁,₁₉ = 9.7, *p* = 0.006), indicating that ΔTAHC values were higher at month 6 than at month 3 across treatment arms. No significant effect of treatment (F₂,₂₀ = 0.1, *p* = 0.951) and no significant time × treatment interaction (F₂,₁₉ = 1.9, *p* = 0.171) were detected. These findings indicate an overall time-associated increase in ΔTAHC, without evidence of differential effects between the tested latanoprost acid concentrations. Descriptive observed values are shown in Fig. 1A and summarized in Table 2. As an exploratory categorical analysis of the primary endpoint, responder rates were calculated from individual ΔTAHC values at month 6. Using a liberal responder definition of any increase in TAHC from baseline, responder rates were 100% in the 0.01% arm, 90.9% in the 0.05% arm, and 83.3% in the 0.1% arm. Using a more conservative threshold of at least 10% increase in TAHC, responder rates were 80%, 72.7%, and 66.7%, respectively. Fisher’s exact tests showed no significant association between treatment arm and responder status for either definition: any increase, *p* > 0.999; at least 10% increase, *p* > 0.999. These exploratory categorical findings should be interpreted cautiously because of the small overall sample size and unequal treatment-arm sizes (Supplementary Material 1, Supplementary Table S3).

#### Supportive analysis: percent change in target-area hair count (%TAHC)

To complement the primary analysis of absolute TAHC change, individual hair-count changes were also analyzed as percent change from baseline. A mixed-effects model for repeated measures (REML) revealed a significant effect of time (F₁,₁₉ = 7.4, *p* = 0.013), indicating that %TAHC values were higher at month 6 than at month 3 across treatment arms. No significant effect of treatment (F₂,₂₀ = 0.3, *p* = 0.77) and no significant time × treatment interaction (F₂,₁₉ = 0.8, *p* = 0.484) were detected. These findings indicate an overall time-associated increase in %TAHC, without evidence of differential effects between the tested latanoprost acid concentrations. Descriptive observed values are shown in Fig. 1B and summarized in Table 2. Overall, the ΔTAHC and %TAHC analyses indicate a time-associated increase in TAHC during latanoprost acid treatment, without evidence of statistically significant between-arm differences within the tested concentration range (Fig. 1A, B).

#### Secondary and exploratory outcomes: trichoscopic measures

Secondary and exploratory trichoscopic measures included yellow-dot counts, a feature linked to inactive/kenogen-phase follicles, and FU composition, assessed here as an indicator of follicular remodeling.

##### Yellow dots

**Fig. 2:**
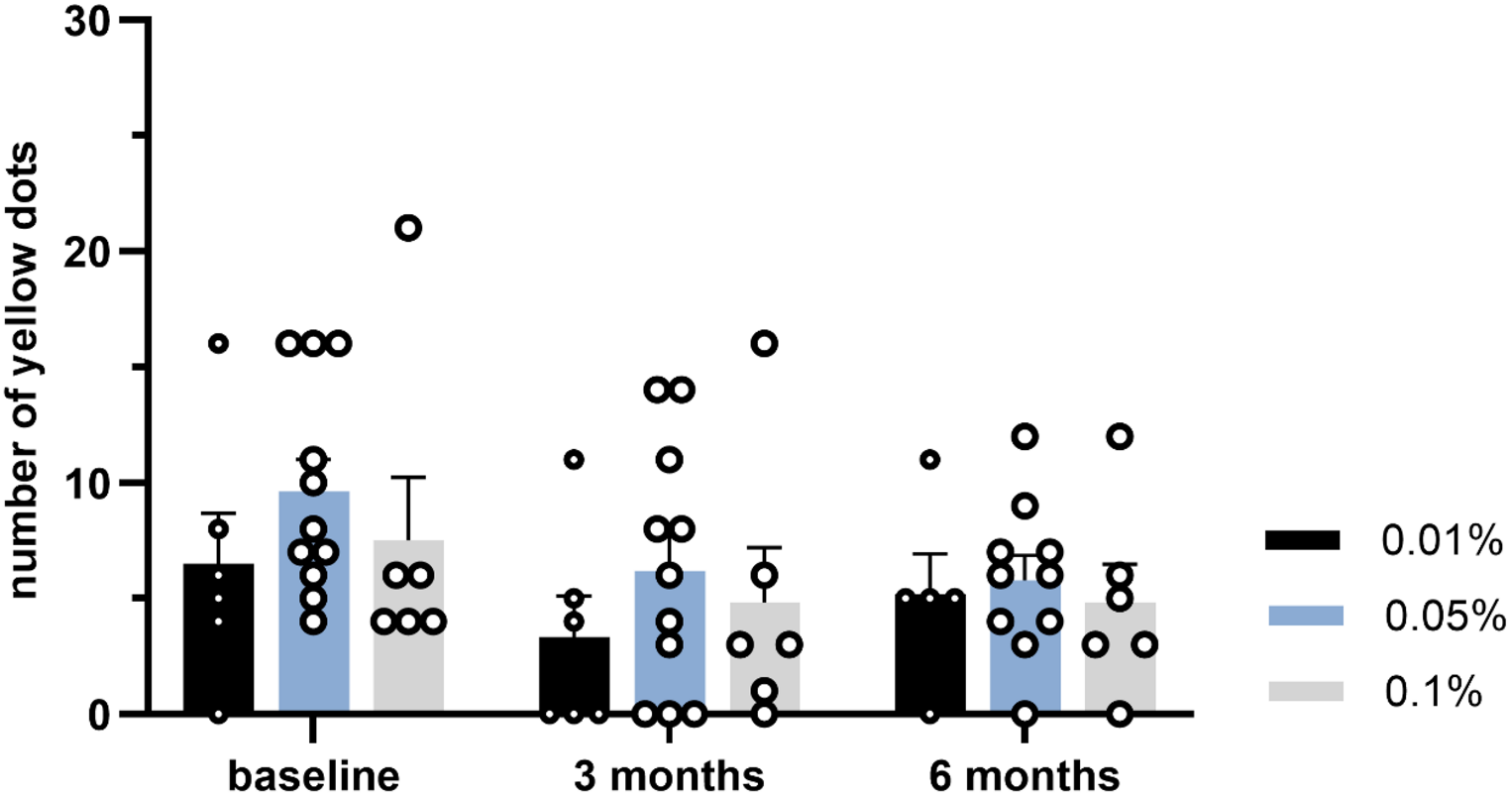
Changes in yellow-dot counts from baseline after 3 and 6 months of treatment with latanoprost acid at concentrations of 0.01%, 0.05%, or 0.1% in women with female androgenetic alopecia¹. Yellow dots were counted using standardized trichoscopy images obtained at fixed scalp locations at baseline, month 3, and month 6. Analyses were performed in participants with available post-baseline assessments: 0.01% (*n* = 6 at baseline and month 3; *n* = 5 at month 6), 0.05% (*n* = 11 at baseline and month 3; *n* = 10 at month 6), and 0.1% (*n* = 6 at baseline, month 3, and month 6). Results are presented as observed means ± SEM. Each point represents data from an individual patient. Statistical analysis: mixed-effects model for repeated measures (REML). Abbreviations: REML, restricted maximum-likelihood; SEM, standard error of the mean

For yellow-dot counts, a mixed-effects model for repeated measures (REML) revealed a significant effect of time (F₂,₃₈ = 15.1, *p* < 0.001), indicating an overall change over the study period across treatment arms. No significant effect of treatment (F₂,₂₀ = 0.5, *p* = 0.593) and no significant time × treatment interaction (F₄,₃₈ = 0.6, *p* = 0.656) were detected. Thus, yellow-dot counts changed over time during topical latanoprost acid treatment, without evidence of concentration-specific effects. Descriptive observed values are shown in Fig. 2 and summarized in Table 2.

¹Investigator-initiated, randomized, double-blind, single-center, dose-ranging pilot trial conducted in 2015–2016 at the Department of Dermatology, Medical University of Warsaw, Warsaw, Poland.

##### FU arrangement

**Fig. 3:**
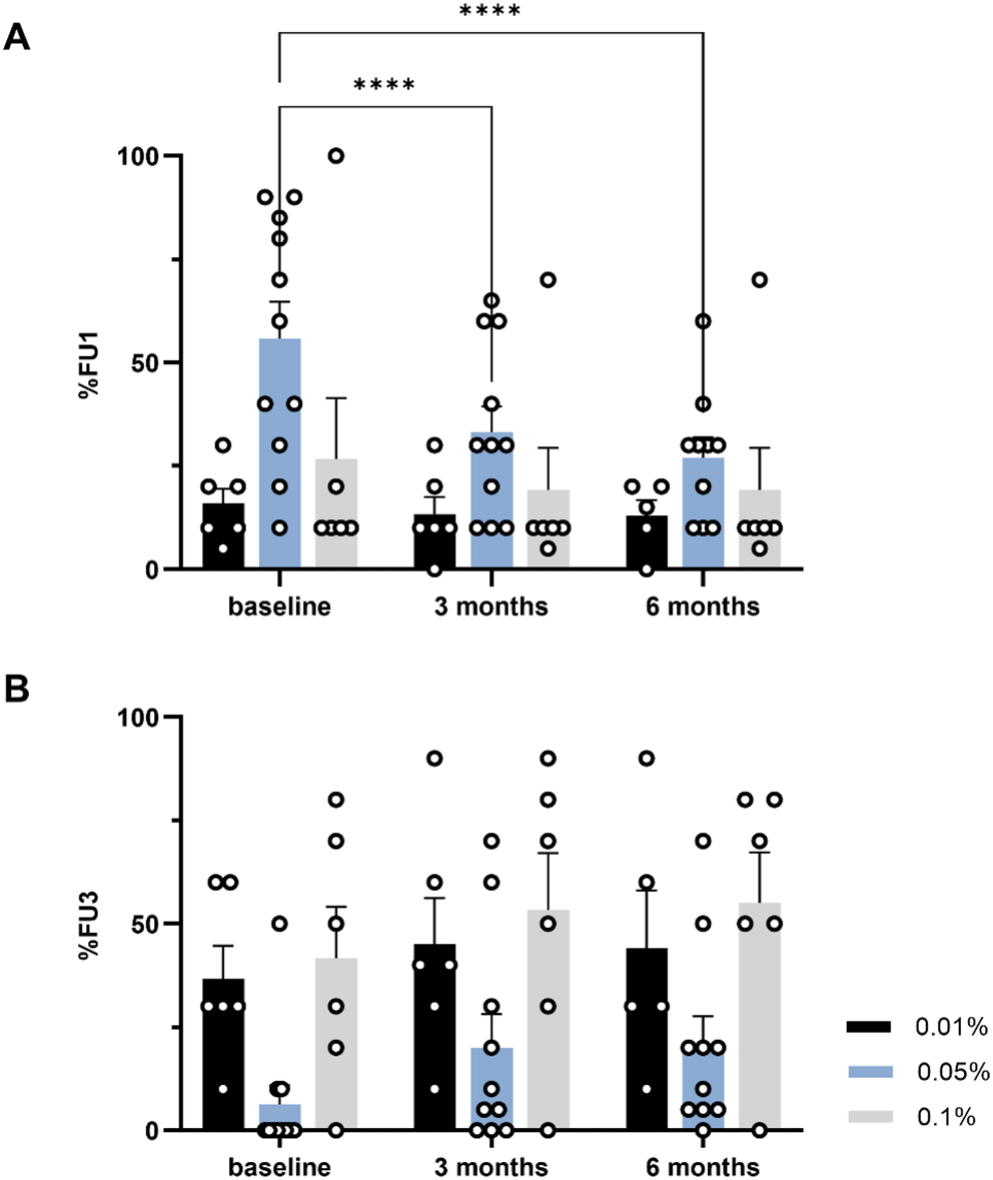
Changes in FU composition from baseline after 3 and 6 months of treatment with latanoprost acid at concentrations of 0.01%, 0.05%, or 0.1% in women with female androgenetic alopecia¹. FU composition was assessed using standardized trichoscopy images obtained at fixed scalp locations at baseline, month 3, and month 6. (**A**) %FU1. (**B**) %FU3. Analyses were performed in participants with available post-baseline assessments. For %FU1: 0.01% (*n* = 6 at baseline and month 3; *n* = 5 at month 6), 0.05% (*n* = 11 at baseline and month 3; *n* = 10 at month 6), and 0.1% (*n* = 6 at baseline, month 3, and month 6). For %FU3: 0.01% (*n* = 6 at baseline and month 3; *n* = 5 at month 6), 0.05% (*n* = 11 at baseline; *n* = 10 at month 3 and month 6), and 0.1% (*n* = 6 at baseline, month 3, and month 6). Results are presented as observed means ± SEM. Each point represents data from an individual patient. In panel A, brackets indicate Tukey-adjusted post hoc comparisons versus baseline for the 0.05% arm; ****p < 0.0001. Statistical analysis: mixed-effects model for repeated measures (REML) with Tukey’s post hoc test. Abbreviations: FU, follicular unit; REML, restricted maximum-likelihood; SEM, standard error of the mean; %FU1, percentage of single-hair follicular units; %FU3, percentage of triple-hair follicular units. ¹Investigator-initiated, randomized, double-blind, single-center, dose-ranging pilot trial conducted in 2015–2016 at the Department of Dermatology, Medical University of Warsaw, Warsaw, Poland

To determine whether the increase in TAHC was accompanied by changes in FU composition, the percentages of single-hair (%FU1) and triple-hair FUs (%FU3) were analyzed at baseline, month 3, and month 6. For %FU1, a mixed-effects model for repeated measures (REML) revealed a significant effect of time (F₂,₃₈ = 12.1, *p* < 0.001), no significant effect of treatment (F₂,₂₀ = 2.8, *p* = 0.083), and a significant time × treatment interaction (F₄,₃₈ = 5.3, *p* = 0.002). These findings indicate that %FU1 changed over time and that the temporal pattern differed between treatment arms. Post hoc comparisons showed that, in the 0.05% arm, %FU1 decreased from baseline to month 3 (model-estimated mean difference, 22.73% points; 95% CI, 12.69 to 32.77; Tukey-adjusted *p* < 0.001) and from baseline to month 6 (31.56% points; 95% CI, 21.16 to 41.96; Tukey-adjusted *p* < 0.001; Fig. 3A; Table 2).

For %FU3, a mixed-effects model for repeated measures (REML) revealed a significant effect of time (F₂,₃₇ = 10.1, *p* < 0.001), a significant effect of treatment (F₂,₂₀ = 4.2, *p* = 0.03), but no significant time × treatment interaction (F₄,₃₇ = 0.8, *p* = 0.538; Fig. 3B). These findings indicate that %FU3 changed over time and differed across treatment arms overall, without evidence that the temporal pattern differed by dose. Numerical baseline variation in FU composition should be considered when interpreting this exploratory endpoint (Fig. 3B; Table 2; baseline characteristics in Table 1).

Exploratory baseline-adjusted sensitivity analyses were performed for month 6 efficacy outcomes to assess the influence of baseline values on follow-up results. After adjustment for the corresponding baseline value, treatment arm was not significantly associated with month 6 TAHC, yellow-dot counts, %FU1, or %FU3. Baseline covariate slopes were below 1 for yellow-dot counts and %FU1 and close to 1 for TAHC; the %FU3 sensitivity analysis should be interpreted cautiously because model assumptions were not fully met (Supplementary Material 1, Supplementary Table S4).

Baseline values shown in Table 2 reflect the endpoint-specific analysis sets used for the corresponding longitudinal analyses and may therefore differ from the baseline values in Table 1, which summarizes all randomized participants with available baseline data.

**Table 2:** Efficacy of latanoprost acid therapy in women with female androgenetic alopecia^1^

Endpoint	Visit	Latanoprost acid treatment arms		
		0.01%	0.05%	0.1%
ΔTAHC, hairs/cm²	Month 3	11.3 ± 3.5 (*n* = 6)	12.1 ± 3.9 (*n* = 11)	15.2 ± 10.3 (*n* = 6)
ΔTAHC, hairs/cm²	Month 6	17.8 ± 4.3 (*n* = 5)	23.5 ± 6.1 (*n* = 11)	16.5 ± 6.5 (*n* = 6)
%TAHC	Month 3	6.9 ± 2.4 (*n* = 6)	9.8 ± 3.6 (*n* = 11)	10.4 ± 6 (*n* = 6)
%TAHC	Month 6	10.4 ± 2.9 (*n* = 5)	19 ± 5.7 (*n* = 11)	13.9 ± 6.9 (*n* = 6)
Yellow dots, count	Baseline	6.5 ± 2.2 (*n* = 6)	9.6 ± 1.4 (*n* = 11)	7.5 ± 2.7 (*n* = 6)
Yellow dots, count	Month 3	3.3 ± 1.8 (*n* = 6)	6.2 ± 1.6 (*n* = 11)	4.8 ± 2.4 (*n* = 6)
Yellow dots, count	Month 6	5.2 ± 1.7 (*n* = 5)	5.8 ± 1.1 (*n* = 10)	4.8 ± 1.7 (*n* = 6)
%FU1	Baseline	15.8 ± 3.7 (*n* = 6)	55.9 ± 8.8 (*n* = 11)	26.7 ± 14.8 (*n* = 6)
%FU1	Month 3	13.3 ± 4.2 (*n* = 6)	33.2 ± 6.3 (*n* = 11)	19.2 ± 10.2 (*n* = 6)
%FU1	Month 6	13 ± 3.7 (*n* = 5)	27 ± 5 (*n* = 10)	19.2 ± 10.2 (*n* = 6)
%FU3	Baseline	36.7 ± 8 (*n* = 6)	6.4 ± 4.5 (*n* = 11)	41.7 ± 12.5 (*n* = 6)
%FU3	Month 3	45 ± 11.2 (*n* = 6)	20 ± 8.1 (*n* = 10)	53.3 ± 13.8 (*n* = 6)
%FU3	Month 6	44 ± 14 (*n* = 5)	20.5 ± 7.1 (*n* = 10)	55 ± 12.3 (*n* = 6)

Women with hair loss predominantly consistent with female androgenetic alopecia received topical latanoprost acid 0.01%, 0.05%, or 0.1% once daily for 6 months. Trichoscopic efficacy outcomes were assessed at baseline, month 3, and month 6; change-from-baseline outcomes are shown for month 3 and month 6. Data are shown as observed mean ± SEM. Statistical analyses were performed using mixed-effects models for repeated measures (REML)

^1^Investigator-initiated, randomized, double-blind, single-center, dose-ranging pilot trial conducted in 2015–2016 at the Department of Dermatology, Medical University of Warsaw, Warsaw, Poland

Abbreviations: FU, follicular unit; REML, restricted maximum-likelihood; n, number of participants; SEM, standard error of the mean; TAHC, target-area hair count; %FU1, percentage of single-hair follicular units; %FU3, percentage of triple-hair follicular units; %TAHC, percent change in target-area hair count; ΔTAHC, absolute change in target-area hair count

#### Hair-shaft thickness improvement grade (0–3)

**Fig. 4:**
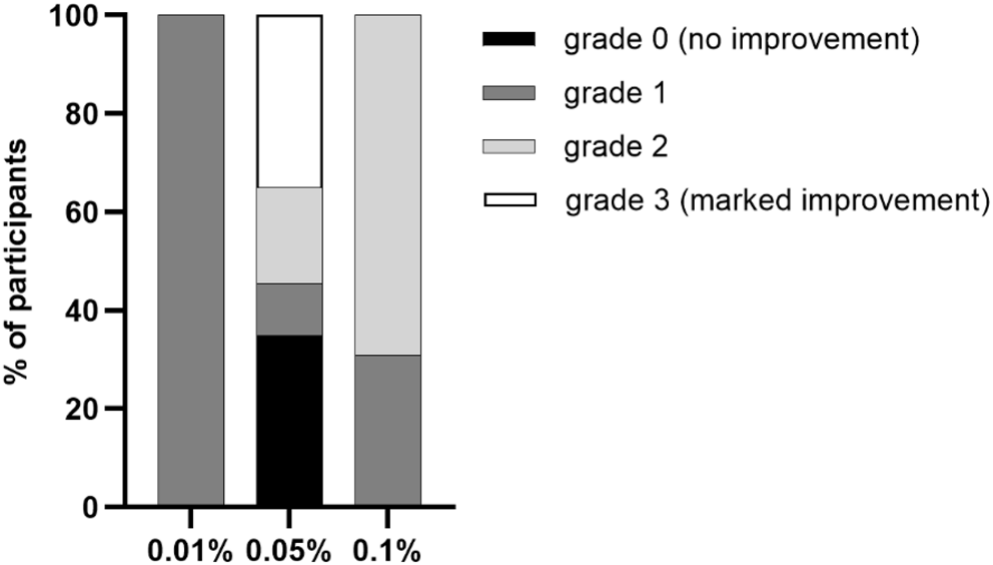
Investigator-rated hair-shaft thickness improvement at month 6 in women with hair loss predominantly consistent with female androgenetic alopecia treated with topical latanoprost acid 0.01%, 0.05%, or 0.1%¹. Hair-shaft thickness was rated by an investigator on an ordinal scale (0 = no improvement; 1 = mild; 2 = moderate; 3 = marked improvement) at month 6. Stacked bars show the percentage of participants in each treatment arm assigned to each improvement grade: 0.01% (*n* = 5), 0.05% (*n* = 11), and 0.1% (*n* = 6). Statistical analysis: Kruskal–Wallis test. ¹Investigator-initiated, randomized, double-blind, single-center, dose-ranging pilot trial conducted in 2015–2016 at the Department of Dermatology, Medical University of Warsaw, Warsaw, Poland

At month 6, investigator-rated improvement in hair-shaft thickness did not differ significantly across latanoprost acid treatment arms (Kruskal–Wallis test, H = 3.279, *p* = 0.188; Fig. 4). The distribution of improvement grades was consistent with modest clinician-rated improvement without evidence of a dose-dependent difference (Fig. 4).

**Fig. 5:**
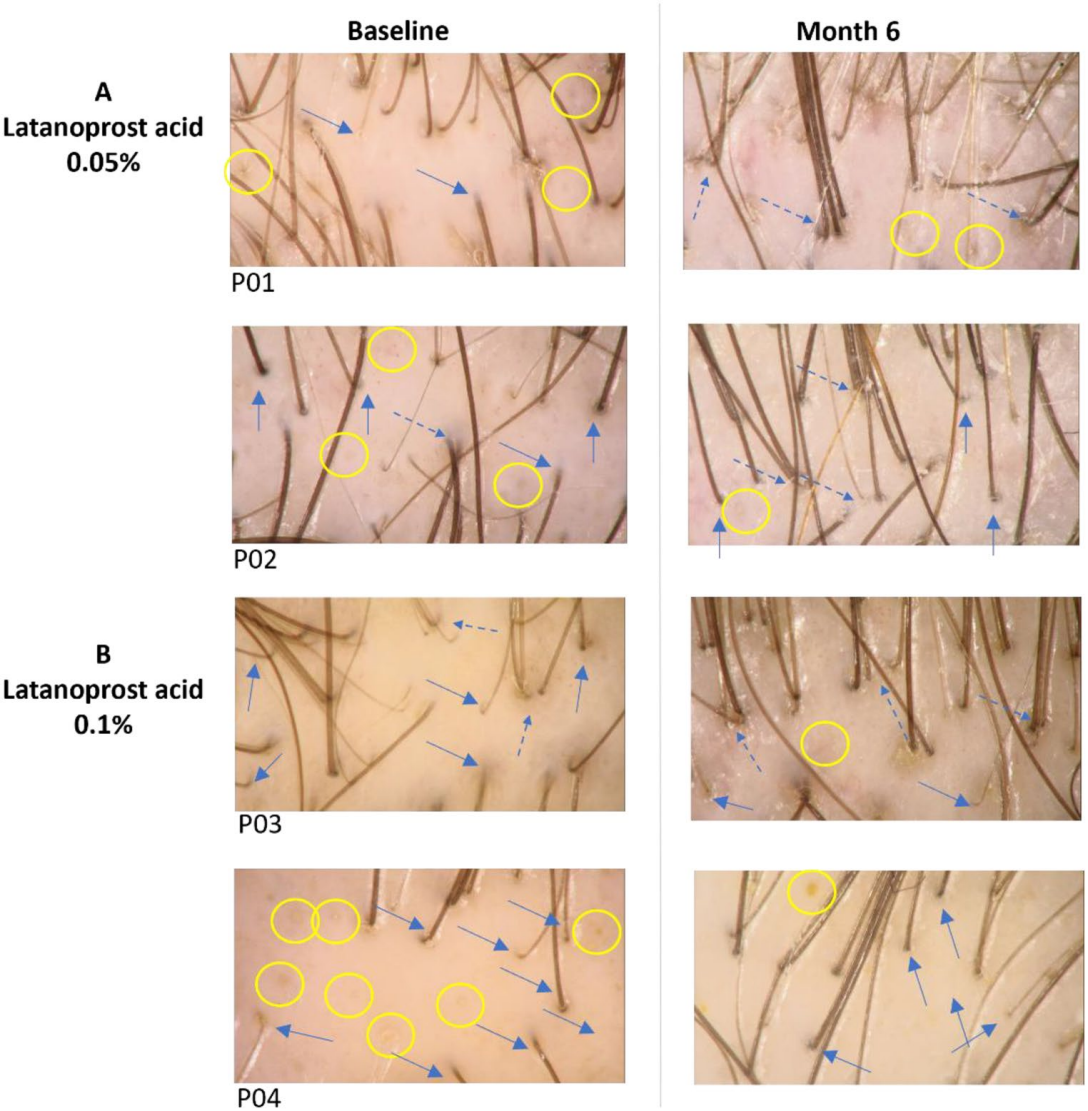
Representative trichoscopic images at baseline and month 6 in women with hair loss predominantly consistent with female androgenetic alopecia treated with latanoprost acid at concentrations of 0.05% or 0.1%¹. Images were obtained from the androgen-dependent scalp region using a standardized acquisition protocol at fixed scalp locations. Baseline and month 6 trichoscopic images (70× magnification) are shown side-by-side for each participant. (**A**) Two representative participants treated with 0.05% (P01, P02). (**B**) Two representative participants treated with 0.1% (P03, P04). P01–P04 denote anonymized participant identifiers. Yellow circles indicate representative yellow dots. Solid blue arrows indicate representative single-hair follicular units (FU1), and dashed blue arrows indicate representative triple-hair follicular units (FU3). The annotations are intended to guide interpretation of the trichoscopic features corresponding to the selected quantitative endpoints reported in Figs. 2 and 3.¹Investigator-initiated, randomized, double-blind, single-center, dose-ranging pilot trial conducted in 2015–2016 at the Department of Dermatology, Medical University of Warsaw, Warsaw, Poland

Representative trichoscopic images from participants in the 0.05% and 0.1% arms at baseline and month 6 are shown in Fig. 5.

#### Patient-reported outcomes

Self-assessed overall improvement was recorded at month 3 and month 6 and analyzed as reported improvement versus no evident improvement (Supplementary Material 1, Supplementary Table S1). At month 3, improvement was reported by 3 of 6 participants in the 0.01% arm, 9 of 11 in the 0.05% arm, and 5 of 6 in the 0.1% arm. At month 6, improvement was reported by 2 of 5, 10 of 11, and 4 of 6 participants, respectively. Fisher’s exact test showed no statistically significant association between treatment arm and self-assessed improvement status at month 3 (*p* = 0.466) or month 6 (*p* = 0.088). These exploratory questionnaire-based findings should be interpreted as supportive descriptive observations rather than confirmatory efficacy outcomes.

#### Safety

No serious adverse events or hospitalizations were reported during the study. Targeted scalp examinations did not reveal clinically relevant local irritation or inflammation, and vital signs remained within normal limits. Two participants discontinued because of reported symptoms: headache in one participant in the latanoprost acid 0.01% arm and increased shedding in one participant in the latanoprost acid 0.05% arm. Additionally, three participants were lost to follow-up after the baseline visit. No formal between-arm statistical comparisons were performed for safety outcomes because of the small number of reported events. Safety and tolerability outcomes were summarized descriptively for all treated participants and are provided in Supplementary Material 1, Supplementary Table S2.

#### In vitro findings

To explore whether the clinical pattern of improvement is consistent with FP receptor-mediated signaling, we compared latanoprost acid and latanoprost in cultured HHDPCs, assessing intracellular Ca²⁺ flux and proliferation-related readouts.

#### FP receptor engagement in HHDPCs (Ca²⁺ flux)

**Fig. 6:**
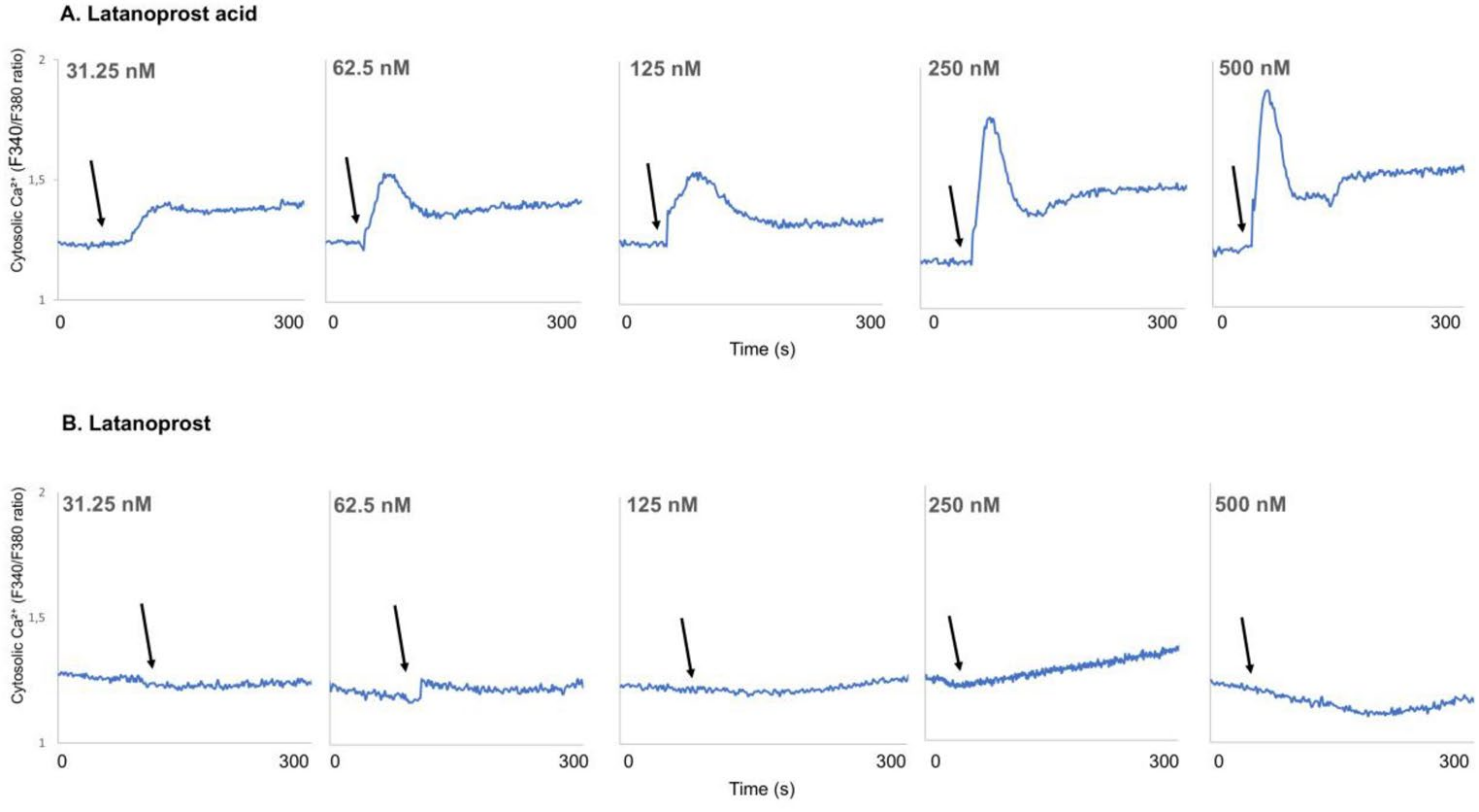
Intracellular Ca²⁺ flux responses to latanoprost acid and latanoprost in HHDPCs. Ratiometric fluorescence (F340/F380) was recorded after Fura-2 AM loading using a spectrofluorimeter. (**A**) Representative traces following the addition of latanoprost acid (31.25–500 nM). (**B**) Representative traces following the addition of latanoprost (31.25–500 nM). Arrows indicate the time point of compound addition. Traces are representative of three independent experiments with comparable results. Abbreviations: HHDPCs, human hair dermal papilla cells

To assess FP receptor-linked signaling in HHDPCs, intracellular Ca²⁺ dynamics were evaluated after exposure to equimolar concentrations of latanoprost acid or latanoprost. Representative Ca²⁺-flux traces showed qualitatively different kinetic profiles after exposure to latanoprost acid and latanoprost. Latanoprost acid produced a rapid, transient increase in the F340/F380 ratio, with a distinct peak followed by a return toward baseline; peak height appeared greater at higher concentrations. In contrast, equimolar latanoprost produced only weak, gradually changing traces without a distinct transient peak under the same experimental conditions. This trace-based pattern is consistent with FP receptor-linked intracellular Ca²⁺ mobilization by the free-acid form, whereas the ester prodrug did not produce a comparable acute Ca²⁺ signal in this assay (Fig. 6).

#### Cell proliferation and metabolic activity in HHDPCs

**Fig. 7:**
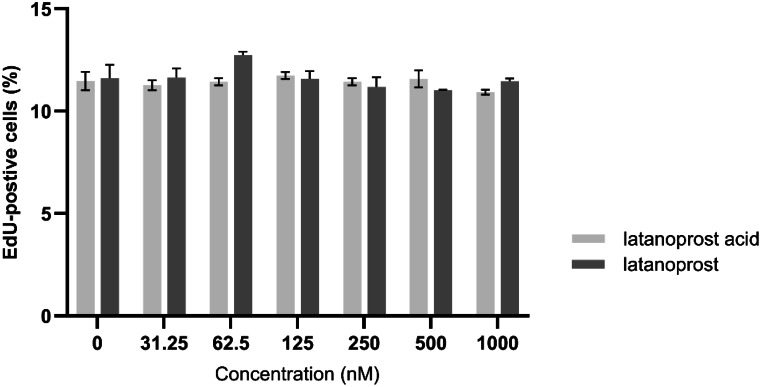
Effect of latanoprost acid and latanoprost on DNA synthesis in HHDPCs. HHDPCs were exposed to latanoprost acid or latanoprost for 48 h. DNA synthesis was assessed using EdU incorporation assay. The percentage of EdU-positive nuclei among total Hoechst-positive nuclei is shown as observed mean ± SEM. Data are shown from three independent experiments; for each experiment, values represent means of technical replicates averaged before statistical analysis. Statistical analysis: for each compound, concentration series were analyzed using one-way ANOVA. Abbreviations: ANOVA, analysis of variance; EdU, 5-ethynyl-2′-deoxyuridine; HHDPCs, human hair dermal papilla cells; SEM, standard error of the mean

To assess whether FP receptor-linked signaling was accompanied by proliferation-related cellular responses, EdU incorporation and PrestoBlue™ metabolic activity were evaluated in HHDPCs after exposure to latanoprost acid or latanoprost. Across the 24, 48, and 72 h exposure periods, neither compound altered the proportion of EdU-positive nuclei compared with vehicle control (0 nM). For the representative 48 h dataset shown in Fig. 7, one-way ANOVA showed no significant effect of treatment condition for either latanoprost acid (F₆,₁₄ = 0.8, *p* = 0.562) or latanoprost (F₆,₁₄ = 2.0, *p* = 0.131). Consistently, neither compound reduced PrestoBlue™ signal compared with vehicle across the tested concentration range (data not shown), supporting the absence of detectable cytotoxicity under these conditions. These findings indicate that latanoprost acid and latanoprost did not induce detectable DNA synthesis or impair metabolic activity in HHDPCs under the experimental conditions tested.

## Discussion

In this investigator-initiated, randomized, double-blind pilot study, topical latanoprost acid was associated with a clinically interpretable trichoscopic bioactivity signal over 6 months in women with female androgenetic alopecia, with a small number of participants with chronic telogen effluvium included. The clearest treatment-arm-specific statistical signal was observed for %FU1, where a significant time × treatment interaction was accompanied by a post hoc reduction in the 0.05% arm. Other trichoscopic endpoints, including ΔTAHC, %TAHC, yellow-dot counts, and %FU3, showed time-associated changes or descriptive patterns consistent with follicular activity, but without statistical evidence of differential temporal effects between the tested latanoprost acid concentrations. Baseline variation in FU composition, together with the small and unequal treatment-arm sizes and attrition, limits dose-related interpretation. Patient-reported overall improvement was recorded across treatment arms, but no statistically significant between-arm differences were detected.

The trichoscopic pattern observed in this study is biologically compatible with improved follicular output, but should be interpreted cautiously. The most statistically supported treatment-arm-specific finding was the reduction in %FU1 in the 0.05% arm, whereas %FU3 and yellow-dot counts showed time-associated changes without evidence of differential temporal effects between the tested concentrations. Taken together, these findings are consistent with a shift away from a single-hair FU pattern and with reduced trichoscopic features linked to inactive/kenogen-phase follicles [20, 21]. However, baseline variation in FU composition, small and unequal treatment-arm sizes, and the exploratory nature of these endpoints limit mechanistic interpretation. Larger confirmatory studies with prespecified imaging and assessment procedures are needed to define whether these trichoscopic changes reflect follicular reactivation, enhanced follicular output, or other changes within the follicular unit.

The present findings do not establish a clear dose–response relationship within the tested concentration range. Although the %FU1 analysis provided the clearest treatment-arm-specific statistical signal, the study was not powered to distinguish between the tested concentrations or to define an optimal dose. Numerical baseline variation, small and unequal treatment-arm sizes, and attrition further limit dose-level interpretation. Exploratory baseline-adjusted sensitivity analyses further supported cautious dose-level interpretation: treatment arm was not significantly associated with month 6 TAHC, yellow-dot counts, %FU1, or %FU3 after adjustment for baseline values. Baseline-covariate slopes below 1 for %FU1 and yellow-dot counts support the possibility that the apparent prominence of the 0.05% arm, in which baseline trichoscopic values were numerically less favorable, partly reflected regression toward the mean rather than a concentration-specific effect. In contrast, the baseline-covariate slope close to 1 for TAHC suggested that the primary hair-count endpoint was less affected by such regression. Potential pharmacodynamic ceiling effects cannot be excluded, but the present pilot data are insufficient to distinguish this possibility from baseline variation and limited statistical power. Therefore, the activity observed within the 0.01% to 0.1% range should be considered hypothesis-generating and requires confirmation in larger, prospectively dose-ranging trials with balanced baseline characteristics and predefined between-arm comparisons. The mechanistic bridge from receptor to clinic is supported on two complementary levels. First, representative Ca²⁺-flux traces in HHDPCs demonstrated that latanoprost acid produced a rapid, transient Ca²⁺ rise, with a distinct peak followed by a return toward baseline; peak height appeared greater at higher concentrations. In contrast, equimolar latanoprost, an ester prodrug, produced only weak, gradually changing traces without a distinct transient peak under the same experimental conditions. This trace-based pattern is consistent with FP receptor-linked intracellular Ca²⁺ mobilization by the free-acid form, whereas the ester prodrug did not produce a comparable acute Ca²⁺ signal in this assay. Second, the trichoscopic profile, including reduced yellow-dot counts and changes in FU composition, is compatible with follicular reactivation and/or increased output from follicles transitioning out of kenonge [19–21]. Prior studies have linked prostaglandin-pathway activation to changes in hair-shaft pigmentation; however, pigmentation was not assessed here and warrants future evaluation [7, 11]. Taken together, these observations support a mechanistically distinct FP receptor-targeted approach that delivers the active free acid, bypassing potential variability in prodrug activation, and provide a coherent biological rationale for clinical translation.

In parallel, EdU incorporation assays demonstrated that neither latanoprost acid nor latanoprost significantly increased DNA synthesis in HHDPCs compared with the corresponding vehicle controls over 24–72 h of exposure across the tested nanomolar concentration range. These findings suggest that FP receptor-linked signaling, as reflected by Ca²⁺-flux traces, was not accompanied by a detectable proliferation-related response in HHDPCs under these experimental conditions. This is consistent with a predominantly signal-modulatory or paracrine role of dermal papilla cells in follicular regeneration rather than direct expansion of the dermal papilla cell population. Accordingly, the clinical and trichoscopic signals observed may reflect enhanced follicular activation and intercellular communication within the follicular niche rather than a primary proliferative mechanism in dermal papilla cells.

Despite long-standing clinical signals that prostaglandin analogues can stimulate hair growth, no prostaglandin-pathway drug is approved for scalp hair loss. To date, bimatoprost 0.03% (LATISSE^®^) remains the only FDA-approved prostaglandin analogue for a hair indication (eyelash hypotrichosis) [22, 23]. A pragmatic contributor to the historical gap in scalp indications may be the prolonged “cosmetic route” in which prostaglandin analogues appeared in over-the-counter eyelash/brow serums in Europe [24, 25], blurring boundaries between cosmetic and medicinal use and dampening incentives for formal drug development. Regulatory scrutiny has tightened only recently. In 2022, the European Union Scientific Committee on Consumer Safety (SCCS) raised explicit safety concerns about prostaglandin analogues in cosmetics [25], and in 2025, the SCCS concluded that specific prostaglandin analogues cannot be considered safe for cosmetic products intended to promote eyelash/eyebrow growth [26]. In parallel, the European Union has advanced updates strengthening prohibitions on unsafe cosmetic substances [27]. Together, these developments have narrowed the cosmetic route and may help realign incentives toward indication-specific medicinal development for scalp hair loss.

Importantly, most clinical work on prostaglandin analogues in scalp hair loss has focused on ester prodrugs rather than free acids, leaving direct delivery of the active moiety underexplored. Addressing this gap, our study is, to our knowledge, the first investigator-initiated clinical evaluation of topically administered latanoprost acid, the active moiety of latanoprost, for scalp hair loss. In this proof-of-concept work, topically applied latanoprost acid exhibited consistent bioactivity signals in human scalp hair, as evidenced by concordant improvements in TAHC and trichoscopic markers over 6 months, alongside a favorable safety profile. Bioactivity was detectable across the tested concentration range, supporting confirmation in larger, prospectively dose-ranging studies.

Given the potential for long-term use, assessing the safety and tolerability of topical latanoprost acid is particularly important. In this study, safety was favorable across concentrations, with no serious adverse events, and targeted scalp examinations did not reveal clinically relevant irritation or inflammation. The absence of systemic clinical signals is consistent with the limited permeability expected for the free-acid form on the scalp and aligns with our a priori safety hypothesis [5, 17]. Nonetheless, longer exposure, larger cohorts, and pharmacokinetic assessment will be necessary to characterize uncommon events and to confirm minimal systemic exposure, particularly if higher concentrations are pursued.

These proof-of-concept data support an adequately powered, randomized, dose-ranging trial across the tested concentration range (0.01% to 0.1%), with PK/PD integration to relate scalp exposure to FP receptor-linked signaling and clinical endpoints. Future studies should extend follow-up to ≥ 12 months to define trajectory and durability; incorporate validated patient-reported outcomes; implement standardized global photography and trichoscopy; evaluate combination therapy (e.g., with minoxidil and/or anti-androgens); consider ex vivo human hair follicle culture as a complementary mechanistic model to further characterize the effects of latanoprost acid on follicular biology and to bridge the gap between cell-based assays and clinical findings; and explicitly contrast prodrug versus free-acid strategies to clarify class effects and optimize translational approaches.

### Limitations

This pilot study was small, single-center, and recruitment was discontinued before the planned sample size was reached. This resulted in unequal treatment-arm sizes and a particularly small vehicle arm, which limited between-arm inference. The 6-month follow-up may not capture maximal response or long-term durability. Although FU metrics were collected at each visit, denominators varied due to attrition, and analyses were sensitive to missingness across time points. Patient-reported outcomes were collected via a structured interview rather than a fully validated instrument. Finally, the inclusion of two participants with chronic telogen effluvium may have contributed to response heterogeneity.

## Conclusions

In this pilot proof-of-concept trial, topical latanoprost acid showed a coherent clinical-trichoscopic bioactivity signal, supported by FP receptor-linked signaling in human hair dermal papilla cells. Because between-dose comparisons were limited by the small overall sample size and unequal treatment-arm sizes, these findings require confirmation in larger randomized pharmacokinetic/pharmacodynamic-integrated trials designed to optimize dose, confirm efficacy, and further characterize long-term safety.

## Supplementary Information

Below is the link to the electronic supplementary material.

Supplementary Material 1

## Data Availability

The data that support the findings of this study are available from the corresponding author upon reasonable request.

## Abbreviations

ANOVA: Analysis of variance
CI: Confidence interval
EdU: 5–ethynyl–2′–deoxyuridine
FAGA: Female androgenetic alopecia
FP receptor: Prostaglandin F2α receptor
FU: Follicular unit
HHDPCs: Human hair dermal papilla cells
n: Number of participants
PD: Pharmacodynamic
PK: Pharmacokinetic
REML: Restricted maximum–likelihood
SCCS: Scientific Committee on Consumer Safety
SD: Standard deviation
SEM: Standard error of the mean
TAHC: Target–area hair count
%FU1: Percentage of single–hair follicular units
%FU3: Percentage of triple–hair follicular units
%TAHC: Percent change in target–area hair count
ΔTAHC: Absolute change in target–area hair count
