# Supplementary material for: Topical latanoprost acid for female androgenetic alopecia: a pilot proof-of-concept trial with mechanistic evidence of prostaglandin F2α receptor activation
Source: Pharmacol Rep. 2026 Jul 10;78(4):1236–49. doi: 10.1007/s43440-026-00876-0 (PMC13437597; doi:10.1007/s43440-026-00876-0)
Supplement: Supplementary file 1 [file 43440_2026_876_MOESM1_ESM.docx]

**Supplementary Material 1**

**Supplementary Table S1. Patient-reported overall improvement in hair condition with topical latanoprost acid in female androgenetic alopecia at months 3 and 6.**

| Visit | Latanoprost acid treatment arms | | | Fisher’s exact test  p value |
| --- | --- | --- | --- | --- |
|  | **0.01%**  **n/N (%)** | **0.05%**  **n/N (%)** | **0.1%**  **n/N (%)** |  |
| Month 3 | 3/6 (50.0%) | 9/11 (81.8%) | 5/6 (83.3%) | p = 0.466 |
| Month 6 | 2/5 (40.0%) | 10/11 (90.9%) | 4/6 (66.7%) | p = 0.088 |

Overall improvement was assessed in women with hair loss predominantly consistent with female androgenetic alopecia who received topical latanoprost acid 0.01%, 0.05%, or 0.1% once daily for 6 months. Responses were dichotomized into reported improvement versus no evident improvement; “yes” or “marked improvement” were counted as improvement, whereas “no”, “do not know”, or “no evident improvement” were counted as no evident improvement. Data are shown as n/N (%), where n is the number of participants reporting improvement and N is the number of participants with available data at the corresponding visit. Statistical analysis: Fisher’s exact test for contingency tables.

**Supplementary Table S2. Safety and tolerability of topical latanoprost acid by treatment arm in female androgenetic alopecia.**

| Treatment arm | Treated participants, n | Serious adverse events or hospitalizations, n | Clinically relevant local scalp findings, n | Discontinuation due to reported symptoms, n | Lost to follow-up after baseline, n |
| --- | --- | --- | --- | --- | --- |
| Vehicle | 2 | 0 | 0 | 0 | 0 |
| Latanoprost acid 0.01% | 8 | 0 | 0 | 1 headache | 2 |
| Latanoprost acid 0.05% | 13 | 0 | 0 | 1 increased shedding | 1 |
| Latanoprost acid 0.1% | 6 | 0 | 0 | 0 | 0 |

Safety and tolerability were assessed in all treated participants who received vehicle or topical latanoprost acid 0.01%, 0.05%, or 0.1% once daily for 6 months. Data are shown as number of participants. Outcomes were summarized descriptively; no formal between-arm statistical comparisons were performed because of the small number of reported events. Vital signs remained within normal limits in all treatment arms.

**Supplementary Table S3. Exploratory responder analysis of topical latanoprost acid based on individual ΔTAHC values at month 6 in female androgenetic alopecia.**

| Responder definition at month 6 | Latanoprost acid treatment arms | | | Fisher’s exact test p value |
| --- | --- | --- | --- | --- |
|  | **0.01% n/N (%)** | **0.05% n/N (%)** | **0.1% n/N (%)** |  |
| Any increase in TAHC from baseline | 5/5 (100.0%) | 10/11 (90.9%) | 5/6 (83.3%) | p > 0.999 |
| ≥10% increase in TAHC from baseline | 4/5 (80.0%) | 8/11 (72.7%) | 4/6 (66.7%) | p > 0.999 |

Responder rates were calculated in participants with available month 6 ΔTAHC data after treatment with topical latanoprost acid 0.01%, 0.05%, or 0.1% once daily for 6 months. Responders were defined using two exploratory thresholds: any increase in TAHC from baseline and at least 10% increase in TAHC from baseline. Data are shown as n/N (%), where n is the number of responders and N is the number of participants with available month 6 ΔTAHC data. Statistical analysis: Fisher’s exact test for contingency tables. Abbreviations: TAHC, target-area hair count; ΔTAHC, absolute change in target-area hair count.

**Supplementary Table S4.** **Baseline-adjusted sensitivity analyses of month 6 efficacy outcomes for topical latanoprost acid in female androgenetic alopecia.**

| Outcome at month 6 | Overall model | Treatment arm adjusted for baseline | Baseline covariate β (95% CI) | Baseline covariate p value | Model R² |
| --- | --- | --- | --- | --- | --- |
| TAHC | F₃,₁₈ = 57.6, p < 0.001 | F₂,₁₈ = 0.3, p = 0.773 | 0.979 (0.813 to 1.144) | p < 0.001 | 0.906 |
| Yellow-dot count | F₃,₁₇ = 7.2, p < 0.001 | F₂,₁₇ = 0.1, p = 0.926 | 0.515 (0.278 to 0.751) | p < 0.001 | 0.561 |
| %FU1 | F₃,₁₇ = 12.2, p = 0.003 | F₂,₁₇ = 1.0, p = 0.376 | 0.507 (0.313 to 0.700) | p < 0.001 | 0.682 |
| %FU3ᵃ | F₃,₁₇ = 16.9, p < 0.001 | F₂,₁₇ = 1.1, p = 0.349 | 1.127 (0.709 to 1.546) | p < 0.001 | 0.749 |

Baseline-adjusted sensitivity analyses were performed using multiple linear regression, with the month 6 value as the dependent variable, the corresponding baseline value as a covariate, and treatment arm as a categorical predictor. Analyses included participants with available baseline and month 6 data for the corresponding endpoint (22 for TAHC; 21 for yellow-dot count, %FU1, and %FU3). The baseline covariate β represents the regression slope relating baseline values to month 6 values after adjustment for treatment arm. ᵃ The %FU3 model should be interpreted cautiously because model diagnostics indicated violation of the homogeneity of regression slopes assumption and non-normal residuals. Abbreviations: CI, confidence interval; FU, follicular unit; TAHC, target-area hair count; %FU1, percentage of single-hair follicular units; %FU3, percentage of triple-hair follicular units.
